# A Marine Actinomycete Rescues *Caenorhabditis elegans* from *Pseudomonas aeruginosa* Infection through Restitution of *Lysozyme 7*

**DOI:** 10.3389/fmicb.2017.02267

**Published:** 2017-11-16

**Authors:** Siti N. Fatin, Tan Boon-Khai, Alexander Chong Shu-Chien, Melati Khairuddean, Amirul Al-Ashraf Abdullah

**Affiliations:** ^1^Centre for Chemical Biology, Universiti Sains Malaysia, Bayan Lepas, Malaysia; ^2^Malaysian Institute of Pharmaceuticals and Nutraceuticals (IPHARM), National Institute of Biotechnology Malaysia, Ministry of Science, Technology and Innovation, Bukit Gambir, Malaysia; ^3^School of Biological Sciences, Universiti Sains Malaysia, Minden, Malaysia; ^4^School of Chemical Sciences, Universiti Sains Malaysia, Minden, Malaysia

**Keywords:** *Caenorhabditis elegans*, *Pseudomonas aeruginosa*, marine actinomycetes, *Streptomyces* sp., *lysozyme 7*

## Abstract

The resistance of *Pseudomonas aeruginosa* to conventional antimicrobial treatment is a major scourge in healthcare. Therefore, it is crucial that novel potent anti-infectives are discovered. The aim of the present study is to screen marine actinomycetes for chemical entities capable of overcoming *P. aeruginosa* infection through mechanisms involving anti-virulence or host immunity activities. A total of 18 actinomycetes isolates were sampled from marine sediment of Songsong Island, Kedah, Malaysia. Upon confirming that the methanolic crude extract of these isolates do not display direct bactericidal activities, they were tested for capacity to rescue *Caenorhabditis elegans* infected with *P. aeruginosa* strain PA14. A hexane partition of the extract from one isolate, designated as *Streptomyces* sp. CCB-PSK207, could promote the survival of PA14 infected worms by more than 60%. Partial 16S sequence analysis on this isolate showed identity of 99.79% with *Streptomyces sundarbansensis*. This partition did not impair feeding behavior of *C. elegans* worms. Tested on PA14, the partition also did not affect bacterial growth or its ability to colonize host gut. The production of biofilm, protease, and pyocyanin in PA14 were uninterrupted, although there was an increase in elastase production. In *lys-7*::GFP worms, this partition was shown to induce the expression of *lysozyme 7*, an important innate immunity defense molecule that was repressed during PA14 infection. GC-MS analysis of the bioactive fraction of *Streptomyces* sp. CCB-PSK207 revealed the presence of methyl esters of branched saturated fatty acids. In conclusion, this is the first report of a marine actinomycete producing metabolites capable of rescuing *C. elegans* from PA14 through a *lys-7* mediated activity.

## Introduction

*Pseudomonas aeruginosa*, an opportunistic human pathogen is a principal cause of nosocomial infection, leading to morbidity and mortality in immune-compromised patients (Moy et al., 2006; Driscoll et al., 2007). Among dangers posed by *P. aeruginosa* is healthcare associated pneumonia and infection of burn patients (Rello et al., 2003; Agodi et al., 2007). The perilous emergence of multidrug resistant *P. aeruginosa* strains is hindering the development and effectiveness of antibiotics (Hauser and Sriram, 2005; Levy, 2005; Aloush et al., 2006). To circumvent problems associated with antibiotic resistance, the search for new anti-infectives targeting bacterial virulence or host immunity have gained momentum (Clatworthy et al., 2007; Hancock et al., 2012). In comparison to traditional antibiotics which exert their effects through bactericidal activities, anti-infectives do not contribute to selection pressure which unwantedly leads to resistance development (Hamill et al., 2008).

The nematode *Caenorhabditis elegans* is readily infected with numerous human bacterial pathogens and amenable to various molecular tools, making it a reliable model for understanding different facets host–pathogen interaction such as, virulence factors and innate immunity pathways (Aballay and Ausubel, 2002). These attributes, coupled with a high degree of conservation with human innate immune signaling pathways, promote the use of *C. elegans* for drug discovery (Artal-Sanz et al., 2006; Burns et al., 2006). The co-existence of both pathogen and host in a host-pathogen relationship provides the capacity of identifying chemical entities capable of rescuing infected host. Academically, this may lead to the discovery of molecules that attenuate bacterial virulence or augment the immunity of the host (Moy et al., 2006). The use of *C. elegans* as in host-pathogen screening assays have been extended to many human pathogens, including *Enterococcus faecalis* (Moy et al., 2006), *Candida albicans* (Breger et al., 2007), *Vibrio alginolyticus* (Durai et al., 2013), *Staphylococcus aureus* (Kong et al., 2014b), *Burkholderia pseudomallei* (Eng and Nathan, 2015), and *Salmonella enteritidis* (Kulshreshtha et al., 2016).

Actinomycetes are persistent soil inhabitants with exceptional capacity to produce clinically useful secondary metabolites, having contributed to more than 50% of the microbial antibiotics discovered (Bérdy, 2005). Early efforts in actinomycetes drug discovery concentrated mostly on soil isolates, due to the erroneous view that the marine environment is a poor source for this group of bacteria (Fenical and Jensen, 2006). However, the diversity of the marine environment enforces a natural selection toward an immeasurable pool of microbial secondary metabolites and may therefore offers a rich and yet unexploited source of actinomycetes, with representatives reported from seawater, intertidal zones, ocean floor, deep ocean trenches, ocean sediments, invertebrates, and plants (Bull et al., 2005). As result, a promising number of novel secondary metabolites with biological properties are constantly being reported from marine actinomycetes (Feling et al., 2003; Lam, 2006; Solanki et al., 2008; Kang et al., 2015). Compounds originating from marine microbes that attenuate virulence through inhibition of quorum sensing system without bacteriocidal activities have also been reported (Fu et al., 2013; Naik et al., 2013).

The *C. elegans*-PA14 relationship has been used to screen natural products from terrestrial plants, endophytic fungi, marine bacteria, and seaweeds, to search for compounds capable of boosting immunity of PA14 infected worms or diminishing quorum sensing and virulence factors (Zhou et al., 2011; Dharmalingam et al., 2012; Kandasamy et al., 2012; Liu et al., 2013). Elsewhere, single compounds such as, curcumin and selenium were also reported to protect *C. elegans* during PA14 infection (Rudrappa and Bais, 2008; Li et al., 2014). Given the vast potential of marine actinomycetes as source of secondary metabolites and the robustness of the *C. elegans*-PA14 screening assay, we utilized the assay to screen for marine actinomycetes capable of producing metabolites that extend the lifespan of infected worms. An extract from *Streptomycetes* sp., conferred survival advantage to the PA14 infected *C. elegans* with a host-directed mechanism partially mediated by the up-regulation of *lys-7* gene. Major compounds in the bioactive fraction were identified as methyl esters of several saturated fatty acids.

## Materials and methods

### Bacteria and worms

PA14 and *Escherichia coli* strain OP50 were cultured as described previously (Dharmalingam et al., 2012). *C. elegans* strain CF4059 with genotype *fer-15*(b26)II; *rol-6*(su1006)II; *fem-1*(hc17)IV which is sterile at 25°C and of the roller phenotype to avoid confounding progeny production during screening and aid in worm scoring were obtained from Cynthia Kenyon Lab (University of California, USA). *C. elegans* strain SAL105 with genotype *pha-1(e2123)* III;denEx2 whose *lys-7* gene was tagged with green fluorescent protein (Alper et al., 2007) were obtained from *Caenorhabditis* Genetics Center (CGC), USA (https://cbs.umn.edu/cgc/home), respectively. Procedures for maintenance and handling of all worms were approved by the Universiti Sains Malaysia Animal Ethics Committee.

### Microbial sample collection

A total of 10 sea bed soil samples were collected from waters at depths ranging from 10 to 20 m deep from Songsong Island, Yan, Kedah, Malaysia (5°48′37.2″N 100°17′47.5″E) on December 2013. The sediment samples were spread on petri plates and dried overnight in laminar flow hood (Valli et al., 2012).

### Isolation of actinomycetes

After drying, samples were heated at 70 ± 2°C for 15 min and were grinded lightly with alcohol-sterilized mortar and pestle. Ten-fold serial dilution up to 10^−5^ was carried out by diluting 1.0 g of sediment sample in 9.0 mL of 50% artificial sea water (ASW). Approximately, 0.1 mL of the mixture was spread on starch casein agar (SCA) supplemented with 80 μg mL^−1^of cycloheximide. All plates were incubated at 28 ± 2°C and observed for actinomycetes growth for 28 days (Mincer et al., 2002; Valli et al., 2012). Grown colonies were observed for morphological differences and listed as candidates for screening assays.

### Preparation of actinomycetes extracts

The isolates were cultured in M1 medium [ingredients: 10.0 g soluble starch, 4.0 g yeast extract, 2.0 g peptone, and 1.0 L distilled water followed by autoclaving at 121°C for 20 min] and incubated with shaking at 28 ± 2°C, 200 rpm for 7–14 days. The culture broth was freeze-dried and extracted with 1:100 (w/v) methanol (MeOH). The mixture was shaken overnight and then filtered using Whatman grade 1 cellulose filter paper with 11 μM pore size. The filtrate was concentrated using rotary evaporator at 60 ± 2°C. The extracts were stored at −20 ± 2°C and adjusted to working concentration with distilled water.

### Anti-microbial assay

Anti-bacterial screening of extracts was performed using the modified Kirby-Bauer disc diffusion method. A few colonies of *P. aeruginosa* PA14 from a culture plate incubated for 24 h were directly inoculated in 0.85% saline. The suspension was compared to a 0.5 McFarland turbidity standard and adjusted with sterile saline. A sterile cotton swab was dipped into the suspension and pressed on the wall of tubes to remove excess bacterial suspension. The swab was repeatedly streaked over the entire surface of Mueller-Hinton agar (Merck, Germany) until the entire surface was streaked. A sterile Whatman antibiotic disc with a 6 mm diameter was placed on the bacterial lawn and 10 μL of 200 μg mL^−1^ crude extract was transferred onto each disc. The plates were inverted and incubated at 37 ± 2°C for 24 h. The zone of inhibition was measured and recorded after the incubation period.

### Slow killing survival assay

For the survival assay, *C. elegans* strain CF4059 was used. Young adult worms were age-synchronized and infected with PA14 on Pseudomonas Infection agar (PIA) as described previously (Dharmalingam et al., 2012). Final concentration of extract in each plate was 200 μg mL^−1^ while a negative control plate contained only distilled water. Worm survival was scored every 24 h, with mortality designated as failure of worm to react by motion when prodded with platinum wire (Powell and Ausubel, 2008). A total time of 96 h was selected as the screening time-point as it gave the best resolution in identifying a potent hit candidate. Worms that crawled onto the plate wall were not included in the final survival analysis.

### Molecular characterization of bacterial isolate

The isolate which produced extract contributing to highest worm survival in the slow killing survival assay described above was cultured in M1 broth for 5 days at 180 rpm at 28 ± 2°C. Genomic DNA of the isolate was extracted using Real Biotech Corporation Hi-Yield Genomic DNA Kit. Universal primers 1492R and 27F were used for the amplification of DNA polymerase chain reaction (PCR) amplification with Applied Biosystem Veriti® 96-Well Fast Thermal. The PCR process involves initial denaturation at 94°C for 3 min followed by 30 cycles of denaturation at 94°C for 30 s, annealing at 55°C for 30 s, extension at 72°C for 60 and 40 s, and final extension at 72°C for 5 min. The PCR product was purified using QIA quick PCR Purification Kit by Qiagen. Sequences of purified DNA samples were aligned with the corresponding phylogenetic tree constructed using MEGA6 (Tamura et al., 2013). Comparison of 16S ribosomal RNA gene sequences of the isolate was done using EzTaxon (Chun et al., 2007).

### Liquid-liquid partitioning of bacterial isolate

The extract of the isolate contributing to highest survival of worms was further partitioned in n-hexane, dichloromethane, ethyl acetate, and butanol. The methanol-aqueous was mixed with each solvent in 1:1 (v/v) ratio in a separator funnel and shaken vigorously. The funnel was let to stand for 15 min and the resulting layers were collected and dried with a rotary evaporator. The subsequent partitions were then employed in a slow killing survival assay. Subsequently, the partition causing highest percentage of worm survival was subjected to the assays described below.

### Dose response assay

The CF4059 worms were exposed to the infection plate as described in the slow-killing assay with or without partition supplementation at 50, 200, 400, and 1,000 μg ml^−1^ final concentration. Worm survival was scored every 24 h.

### Pharyngeal pumping assay

*C. elegans* CF4059 was exposed to the infection plates as described in the slow-killing assay. The pharyngeal pumping of three randomly picked worms was observed for 20 s at 12 h interval using Leica Stereomicroscope M205 FA. Pumping rate was measured by counting grinder movement and contraction/relaxation cycles of the bulb (Hobson et al., 2006).

### Growth of *P. aeruginosa* PA14

Method for kinetic growth study was a modification of an earlier protocol (Hall et al., 2014). A few PA14 colonies from 24 h freshly cultured plates were inoculated in 250 mL conical flask with 50 mL Mueller-Hinton broth (Merck, Germany), followed by incubation at 37°C, 180 rpm for 24 h. The culture was transferred into 50 mL centrifuge tube and centrifuged at 2,775 × *g* for 30 min. The supernatant was discarded and remaining cell pellet was washed twice. The cell pellet was then dissolved in fresh MH broth. The treatment well contained 10 μL of bacterial cell culture, 180 μL of MH broth and 10 μL of 8 mg/mL extract. Control well only contained 10 μL of bacterial cell culture and 190 μL of MH broth. The microtiter plates were incubated in the microplate reader at 37°C for 24 h with sampling interval every 4 h at 625 nm (BioTek Synergy Mx, USA).

### PA14 biofilm assay

Biofilm assay was carried out as described previously (O' Toole, 2011). PA14 was cultured in Luria Bertani (LB) broth overnight at 37°C with shaking at 180 rpm. PA14 was cultured in LB broth at 37°C with 180 rpm shaking overnight. A 96-well biofilm assay plate with 400 μg mL^−1^ of actinomycete partition in LB broth was inoculated with the overnight PA14 culture at 1:100 ratio and further incubated overnight at 37°C. The cells were discarded and plate was rinsed with tap water. Biofilm formed on the wall of the plate was stained with 1% crystal violet and solubilized with 30% acetic acid in water. Optical density was measured using microtiter plate reader (SpectraMax M5) at 550 nm wavelength.

### PA14 protease, elastase assay, and pyocyanin assay

Protease and elastase assay were carried out as described elsewhere (Rudrappa and Bais, 2008). PA14 was cultured at 37°C for 24 h, with or without presence of *Streptomyces* sp. partition in LB broth. The supernatant was collected and filtered using 0.22 μM nylon filter. About 50.0 μL supernatant was added into the reaction mixture consisting of 0.8% azocasein (Sigma) in 500 μL of 50 mM K_2_HPO_4_ at pH7. The reaction mixture was incubated at 25°C for 3 h. The reaction was stopped by adding 0.5 mL of 1.5 M HCl into the mixture. The tubes were placed on ice for 30 min and centrifuged at 7,826 × *g* for 10 min. Finally, 0.5 mL of 1 M NaOH was added into the tubes and the reading was measured at 440 nm.

For elastase assay, 50.0 μL supernatant was added to 1.0 mL of 10 mM Na_2_HPO_4_ at pH7 and 20 mg of elastin-Congo red. The tubes were incubated for 4 h at 37°C with 180 rpm shaking. The tubes were centrifuged at 7,826 × *g* for 10 min and the optical density reading was taken at 495 nm.

Pyocyanin assay was carried out as described elsewhere (Essar et al., 1990). PA14 was cultured with or without presence of *Streptomyces* sp. metabolites and supernatant was collected as above. About 4.5 mL of chloroform was added to 7.5 mL of collected supernatant and vortexed for 20 s. The mixture was centrifuged at 2,880 × *g* for 10 min. About 3.0 mL of the resulting blue layer at the bottom of the tube was transferred into a new tube, followed by addition of 1.5 mL of 0.2 M HCl and vortexing for 20 s. Tubes were then centrifuged for 2 min at 2,880 × *g* and 1.0 mL of the ensuing pink layer was transferred into cuvettes and reading was taken at 520 nm (Thermo Scientific Genesys20).

### Visualization of *lys-7* in *C. elegans*

Slow killing assay was carried out using the transgenic *lys-7*::GFP *C. elegans* strain SAL105. The fluorescence micrograph of worms was captured using a Leica Microsystem M205 FA following 24 h of pathogen exposure. Images were analyzed using Image J (National Institutes of Health, USA) to quantify *lys-7* fluorescent intensity.

### Preparative TLC fractionation

TLC plate (Merck TLC silica gel 60 F_254_, Germany) coated with silica was used as the stationary phase. Sample of hexane partition was prepared by diluting 10 mg of extract in 1.0 mL of CHCl_3_. The sample was spotted on the plates with a capillary tube. The plates were then put in developing chamber with solvent system of methanol:chloroform of 20:80. After drying, a small portion of the plate was cut, followed by staining with vanillin-sulphuric acid reagent (Yadav and Gupta, 2013). The stained plates were then air-dried for 15 min and oven-dried at 96 ± 2°C for 8 min (Maurya and Srivastava, 2013). Spots formed were aligned on the plate and marked. The marked area was scraped using a scalpel. The collected fractions were dissolved in 100% ethanol, filtered by using Whatman no.1 filter paper and rotated to dryness. The fractions were subjected for survival assay and the fraction with positive result was sent for GC-MS analysis (Agilent 6890, USA) with capillary column of 30 m × 0.25 mm × 0.25 μm (Agilent HP-5 ms, USA). The flow rate was set at 1.2 mL/min, with 10 μL sample injection. Helium gas was used and total run was 40 min. The obtained spectrum was compared with NIST Spectral Library for compound identification.

### Data analysis

All numerical data were analyzed using GraphPad Prism 5 and StatView5.0.1 (SAS Institute, Inc) software. Values were presented as mean ± standard deviation (SD) of at least two independent experiments. Data from the killing assays were analyzed with StatView 5.0.1 and plotted using the Kaplan-Meier Cumulative Survival Plot for Time (non-parametric survival analysis). The comparison was analyzed using the GraphPad Prism 5 Log-rank (Mantel-Cox) significance test. Data from dose response assay, pharyngeal pumping assay, biofilm assay, kinetic growth of PA14 and total cell fluorescent count were analyzed with GraphPad Prism 5 unpaired *t*-test.

## Results

### Isolation of marine actinomycetes and antimicrobial assay

A total of 18 morphologically different strains were successfully isolated from the marine sediment samples (Table S1). These isolates produced aerial mycelium, with four of the isolates showing pigmentation. All isolates produced mycelial clump when cultured in broth medium after incubation in shaker at 180 rpm and 28 ± 2°C. All 18 isolates were extracted and subjected to anti-microbial assay. None of these isolates caused a visible inhibition zone on the *P. aeruginosa* PA14 lawn (data not shown) which means these extracts do not possess bactericidal activities toward PA14.

### Effect of marine actinomycetes methanol extracts and partitions on survival of PA14 infected *C. elegans* worms

**Figure 1:**
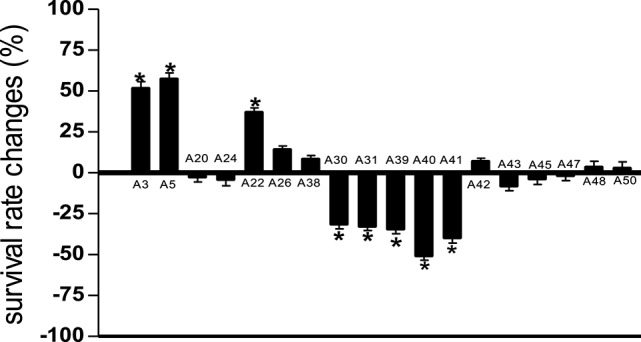
Survival of *C. elegans* infected with *Pseudomonas aeruginosa* PA14 in the presence of marine actinomycetes crude methanolic extract. A5 crude methanolic extract resulted in significantly highest *C. elegans* survival during the PA14 killing assay. ^*^Denotes significance in the Log-rank test in comparison to the untreated control (*p* < 0.05). Data were representative of two independent experiments.

**Figure 2:**
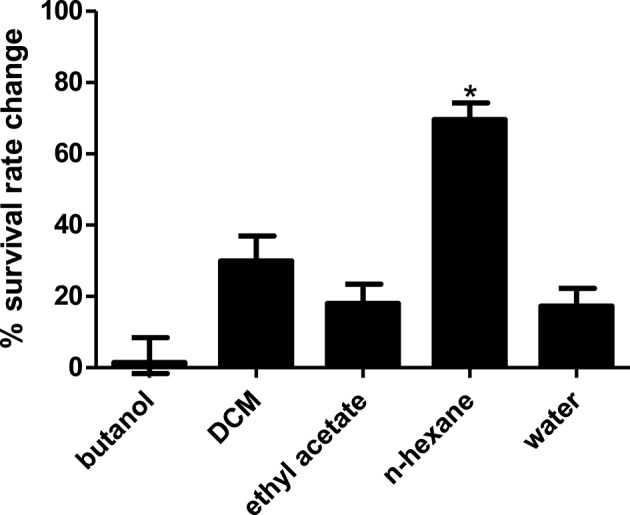
Survival of *C. elegans* infected with *Pseudomonas aeruginosa* PA14 in the presence of different portion of A5 isolate. Hexane partition of the A5 methanolic extract resulted in significantly highest *C. elegans* survival during PA14 killing assay. ^*^Denotes significance in the Log-rank test in comparison to the untreated control (*p* < 0.05). Data were representative of three independent experiments.

Compared to untreated PA14 infected worms, infected worms treated with 8 of the 18 marine actinomycetes extract, respectively, (A3, A5, A22, A26, A38, A42, A48, and A50) showed improved survival rates (Figure 1). Among these, statistically significant increase in survival rate was achieved with A3, A5, and A22 (36.99 ± 2.80–57.31 ± 3.85%), with isolate A5 contributing to the highest survival rate. In tandem, A5 also delayed mortality of infected worms, as represented in TD50 value (Table S2). In addition, worms treated with the remaining extracts (A20, A24, A30, A31, A39, A40, A41, A43, A45, and A47), showed increased susceptibility to killing by PA14. Further partitioning of the isolate A5 methanol extract followed by slow killing assay revealed the hexane partition of isolate A5 to be most potent in attenuating the killing of infected worms, with survival rate of 69.65 ± 4.50% (Figure 2) and TD50 = 102.2 ± 5.54 h (Table S3).

### A5 isolate identified as *Streptomyces* sp. CCB-PSK207

**Figure 3:**
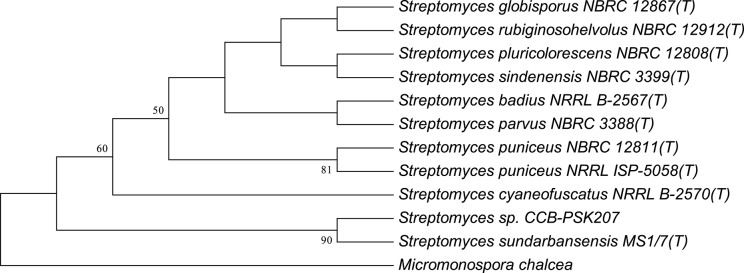
Tamura-Nei model of phylogenetic tree. Tree based on 16S rRNA gene sequences obtained by the Neighbor Joining (NJ) method showing the position of A5 isolate among its phylogenetic neighbors. Numbers at nodes indicate levels of bootstrap support (%) based on a NJ analysis of 1,000 resampled datasets.

Using partial 16S analysis, the A5 isolate showed sequence identity of 99.7–99.85% with several Streptomyces sp. This include *Streptomyces sundarbansensis, Streptomyces puniceus, Streptomyces badius, Streptomyces sidensis, Streptomyces rubiginosohelvolus, Streptomyces pluricolorescense, Streptomyces parvus, Streptomyces globisporus*, and *Streptomyces cyaneofuscatus* (Figure 3). The sequence was deposited in NCBI GenBank under accession number KX372372. On ISP2 agar plates, A5 isolate produced white beige aerial mycelia and brownish substrate mycelia with no pigmentation (Figure S1). A5 isolate was named as Streptomyces sp. CCB-PSK207.

### Effect of *Streptomyces* sp. CCB-PSK207 hexane partition on survival of PA14-infected *C. elegans*

**Figure 4:**
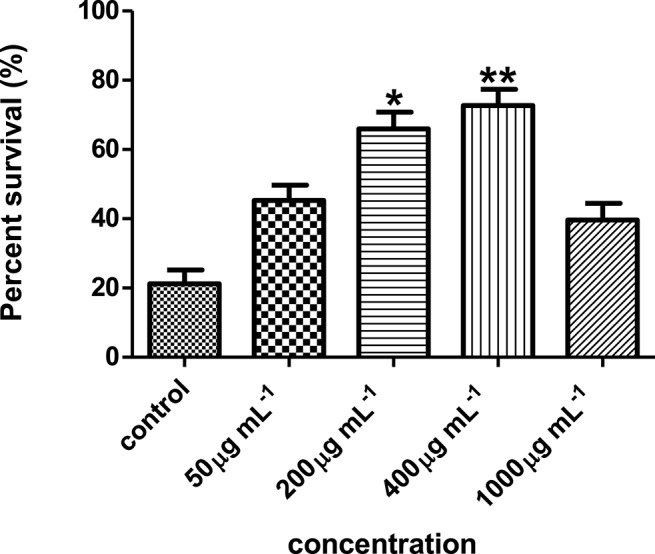
*Streptomyces* sp. CCB-PSK207 hexane partition promotes survivability of PA14-infected worms in a dose dependent manner. Concentration of 400 μg mL^−1^showed the highest percent survival of the worms compared to the untreated control. ^*^(*p*<0.05) and ^**^(*p*< 0.01) denotes statistically significance in Dunnett's test in comparison to the untreated control. Data were representative mean ± SD of three independent screenings at 96 h' time point.

Results showed that the hexane partition of the *Streptomyces* sp. CCB-PSK207 extract promoted survival of PA14-infected worm in a dose dependent manner with a gradual increase observed from 45.33 ± 4.32 to 72.71 ± 4.66% at concentration range of 50–400 μg mL^−1^(Figure 4). There was no further increase in *C. elegans* survival promotion at 1,000 μg mL^−1^ concentration.

### Effect of *Streptomyces* sp. CCB-PSK207 hexane partition on *C. elegans* feeding activities

**Figure 5:**
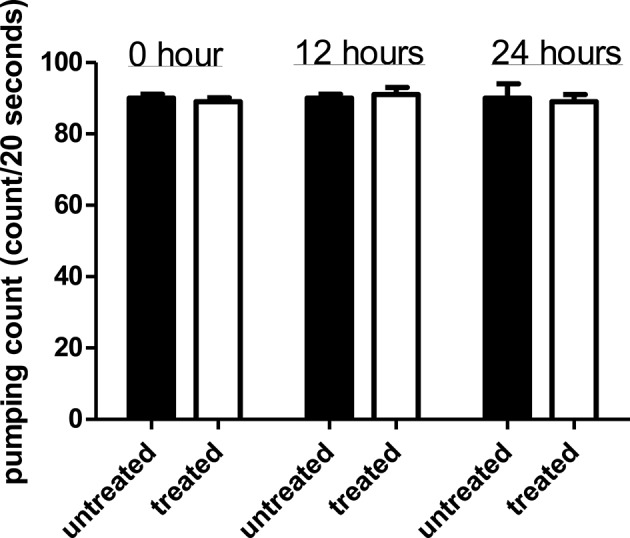
*Streptomyces* sp. CCB-PSK207 hexane partition does not impair *C. elegans* feeding activities. There is no distinguishable difference observed in the pumping rate count between the extract-treated and control worms at the indicated time points (*t*-test, *p*-value of 1.00, 0.392, and 1.00 at 0, 12, and 24 h time-points, respectively). Representative result is depicted from three independent experiments and values are expressed in mean ± SD.

Comparison of pharyngeal pumping rate in *C. elegans* exposed to the *Streptomyces* sp. CCB-PSK207 hexane partition with worms without presence of partition showed no significant difference for all three time points (Figure 5). This indicated that the *Streptomyces* sp. CCB-PSK207 hexane partition did not interrupt *C. elegans* feeding rate.

### Effect of *Streptomyces* sp. CCB-PSK207 hexane partition on PA14 growth

**Figure 6:**
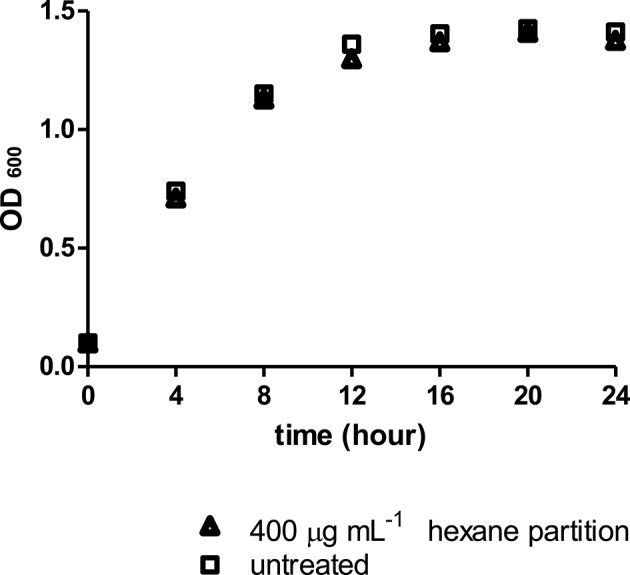
*Streptomyces* sp. CCB-PSK207 hexane partition did not impair PA14 growth at 400 μg mL^−1^. Data were analyzed with unpaired *t-*test, *p* = 0.917. Data are expressed in mean ± SD. Experiments carried out in three independent experiments

Similarly, the *Streptomyces* sp. CCB-PSK207 hexane partition did not impair growth kinetics of PA14, which denote that the rescue of PA14 infected worms did not occur through killing of pathogens (Figure 6).

### Effect of *Streptomyces* sp. CCB-PSK207 hexane partition on PA14 virulence factor production

**Figure 7:**
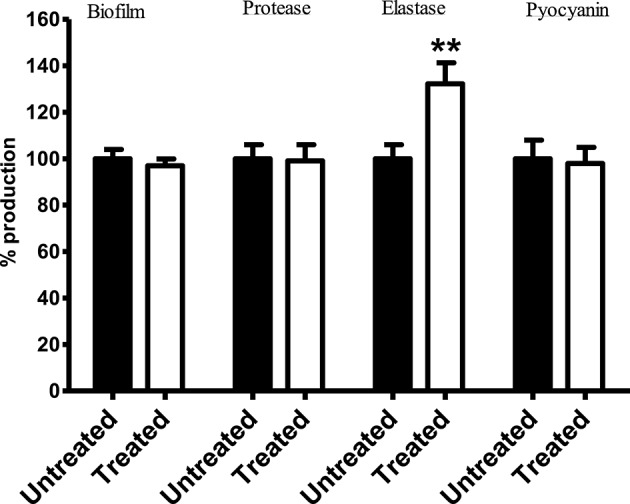
Production of PA14 virulence factor upon treatment with *Streptomyces* sp. CCB-PSK207 hexane partition. There is no distinguishable difference in the production level of biofilm formation (*p* = 0.0530), protease (*p* = 0.0540), and pyocyanin (*p* = 0.0735) between extract-treated and control worms. Significant increase in elastase production level were observed following extract treatment (*p* = 0.0424). All the assays were carried out in three independent experiments. Results are expressed in mean ± SD. Data were analyzed with one sample *t-test* where ^**^denotes statistically significance (*p*<0.01) in comparison to the untreated control.

Results showed no significant difference in the level of biofilm, protease, and pyocyanin production between control PA14 and those exposed to *Streptomyces* sp. CCB-PSK207 hexane partition (Figure 7). However, a significant increase in elastase production was observed in treated PA-14.

### Effect of *Streptomyces* sp. CCB-PSK207 hexane partition on the expression of *lys-7* in PA14 infected *C. elegans*

**Figure 8:**
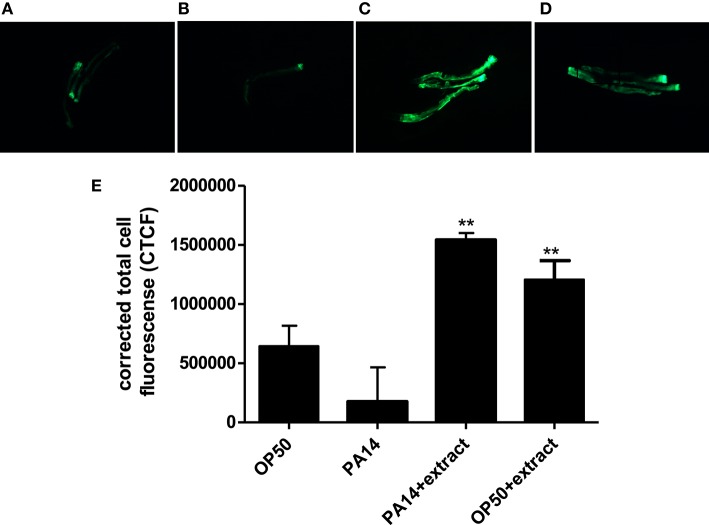
Induced expression of *lys-7* in PA14 infected worms upon treatment with *Streptomyces* sp. CCB-PSK207 hexane partition. Representative fluorescence micrographs of worms after 24-h incubation with partition. **(A)** Worms fed on OP50, uninfected; **(B)** PA14-infected worms without extract treatment and **(C)** PA14-infected worms treated with 400 μgmL^−1^ hexane extract; **(D)** worms fed on OP50 and treated with 400 μgmL^−1^ hexane extract, uninfected. Worms were examined under Leica Microsystem M205 FA with magnification x127. “+” denotes anterior head region of the worms. **(E)** Corrected total cell fluorescence of *lys-7* micrograph A, B, C, and D. Data were analyzed with the Image J software version 1.49. ^**^Denotes significance (*p*<0.01, *t-test*) in comparison to the untreated control.

As compared to worms fed with *E. coli* OP50, PA14-infected worms showed diminished fluorescent signal (Figures 8A,B). However, treatment with *Streptomyces* sp. CCB-PSK207 hexane partition appear to restore GFP expression in both PA 14 infected worms and OP50 fed worms (Figures 8C,D). Imaging-based software quantification showed that worms exposed to *Streptomyces* sp. CCB-PSK207 metabolites produced significantly highest intensity of GFP expression (Figure 8E).

### Effect of *Streptomyces* sp. CCB-PSK207 fraction on survival of PA14-infected *C. elegans*

**Figure 9:**
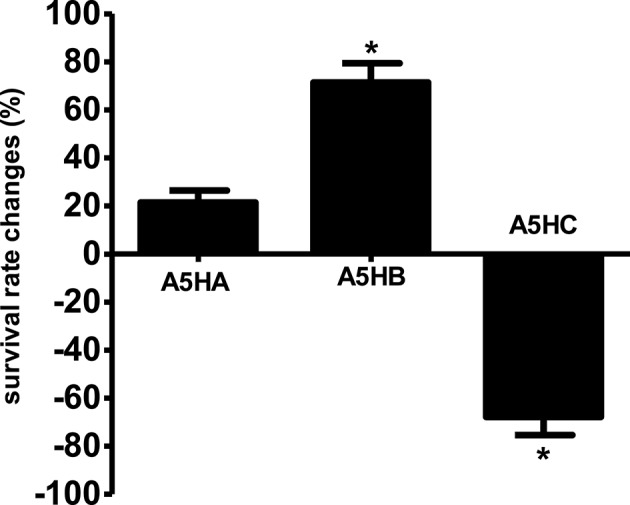
Survival of *C. elegans* infected with *Pseudomonas aeruginosa* PA14 in the presence of different fractions in the killing assay. Fraction A5HB significantly promotes *C. elegans* survival during PA14 killing assay. ^*^Denotes statistically significance in the Log-rank test in comparison to the untreated control (*p* < 0.05). Data were representative of two independent experiments.

Three fractions were collected from preparative TLC and further employed in the *C. elegans* slow killing survival assay. Among them, fraction A5HB showed a significant increase in worm survival rate at 71.43 ± 4.67% (Figure 9) and TD50 of 93.6 ± 1.9 h (Table S4). Fraction A5HA resulted in < 20% worm survival, while A5HC increased the susceptibility of worms toward mortality caused by PA14, respectively.

### Chemical profiling of *Streptomyces* sp. CCB-PSK207 A5HB fraction

Further chemical profiling and compound identification was done using GC-MS (Pollak and Berger, 1996). Seven main compounds were identified, Figure S2 namely (1) tetradecanoic acid methyl ester, (2) pentadecanoic acid 14-methyl methyl ester, (3) tetradecanoic acid, 12-methyl-methyl ester, (4) tridecanoic acid methyl ester, (5) hexadecanoic acid methyl ester (6) octadecanamide, and (7) 1,2-benzenedicarboxylic acid mono(2-ethylhexyl) ester (Table 1).

**Table 1:** Compounds identified from fraction A5HB using GC-MS.

**No**.	**Retention time (min.)**	**Compound**	**Formula**	**Molecular weight (MW)**	**Quality (%)**
1	9.37	Tetradecanoic acid methyl ester	C_15_H_30_O_2_	242	96
2	9.93	Pentadecanoic acid 14-methyl-methyl ester	C_16_H_32_O_2_	256	94
3	9.97	Tetradecanoic acid 12-methyl-methyl ester	C_16_H_32_O_2_	256	89
4	10.45	Tridecanoic acid methyl ester	C_14_H_28_O_2_	228	96
5	10.64	Hexadecanoic acid methyl ester	C_17_H_34_O_2_	270	96
6	11.29	9-octadecanoic acid methyl ester	C_19_H_36_O_2_	296	60
7	13.60	1,2-benzenedicarboxylic acid mono(2-ethylhexyl) ester	C_16_H_22_O_4_	278	90

*Chemical profile of compounds was compared with NIST spectral library*.

## Discussion

Given the multiple health hazards posed by *P. aeruginosa* and the rise of multi-drug resistant strains, it is essential that novel drugs with anti-infective properties are discovered (Hauser and Sriram, 2005). Marine actinomycetes are a promising target, as exemplified by consistent discovery of promising metabolites against fungal, parasitic, bacterial, and viral diseases (Rahman et al., 2010; Subramani and Aalbersberg, 2012; Manivasagan et al., 2013). The *C. elegans*-PA14 slow killing assay has been employed to search for potential immune-boosting metabolites (Adonizio et al., 2008b; Zhou et al., 2011; Durai et al., 2013; Li et al., 2014). We report here the use of this assay to screen marine actinomycetes for anti-infective properties against *P. aeruginosa*.

We first showed that the actinomycetes extracts did not directly inflict mortality on PA14. This will rule out the detection of compounds with bactericidal effects and divert subsequent discovery to isolation of lead compounds targeting immunity of host or virulence of pathogen. Using the slow killing assay, we discovered a partition from the methanol extract of an actinomycetes isolated from sea sediment which significantly boost the survival in PA14-infected worms in a dose dependent manner. This increase is comparable to level of survivals observed in PA14 infected worms treated with *Swietenia macrophylla* seed extract (Dharmalingam et al., 2012) and curcumin (Rudrappa and Bais, 2008). Using 16S analysis, this isolate was shown to have >99.5% identity with several *Streptomyces* sp. and was designated as *Streptomyces* sp. CCB-PSK207. Among all actinomycetes, the *Streptomycetes* group is economically valuable, giving rise to 50–55% of known antibiotics (Bérdy, 2005). However, only a small portion of marine actinomycetes have been subjected for bioprospecting of new therapeutics. Besides efficacy, having the host in the screening assay provides an added advantage of early indication of compound toxicity (Squiban and Kurz, 2011). This could plausibly explain the higher mortalities encountered by worms exposed to several of the crude methanol extracts in this present study.

Slow killing of *C. elegans* by PA14 involves the colonization and proliferation of pathogen in the host gut (Tan et al., 1999a). As such, it is important to establish if *Streptomyces* sp. CCB-PSK207 metabolites mitigate killings by diminishing gut colonization in worms. Since the colonization of PA14 in nematode gut commence with feed intake, a reliable indicator is observation of the pumping rate of *C. elegans* pharynx, a tube involved in feeding and transportation of bacteria into the gut (Avery and Shtonda, 2003). Overall, our results showed that PA14 exposed to *Streptomyces* sp. CCB-PSK207 partition could still grow and colonize gut of host after feeding. These observations were also reported with anti-infective natural products isolated from a similar screening approach (Rudrappa and Bais, 2008; Dharmalingam et al., 2012; Durai et al., 2013; Kong et al., 2014a,b).

A plausible scenario to explain the improved survival of infected worms treated with the bioactive partition is the presence of compounds with anti-virulence activities. The widespread problems associated with PA14 is principally due to the production of a series of virulence factors including protease, elastase, pyocyanin, and alginate (Lyczak et al., 2000). In addition, formation of obdurate biofilms is a crucial armory in PA14's persistency against antimicrobial therapy (Ma et al., 2009). During PA14 infection of *C. elegans*, the pathogens produce virulence-related membrane vesicles, leading to the accretion of biofilm-like material on host intestinal cells (Irazoqui et al., 2010). There have been several reports on natural product-based small molecules from marine organisms, including actinomycetes showing potency against virulence of PA14 (Hentzer et al., 2003; Fu et al., 2013; Naik et al., 2013; Yaniv et al., 2017). Our results showed that *Streptomyces* sp. CCB-PSK207 hexane partition did not subdue production of biofilm, protease, and pyocyanin in PA14. This supports our earlier observation of normal gut colonization in nematode exposed to the bioactive extract as PA14 mutants with perturbed quorum sensing cascade are unable to colonize the gut of *C. elegans* (Tan et al., 1999b). Extracts of several terrestrial and aquatic plant species, have been reported to rescue *C. elegans* from mortality by interfering with PA14 quorum sensing and virulence activities (Adonizio et al., 2008a,b; Rudrappa and Bais, 2008; Kandasamy et al., 2012; Husain et al., 2013; Liu et al., 2013; Sarabhai et al., 2013). Therefore, a disparity between these chemical entities and *Streptomyces* sp. CCB-PSK207 is that the latter did not seemed to rescue PA14 infected worms through disruption of pathogen virulence factors. Elsewhere, similar to our results, selenite did not reduce both quorum-sensing signals and virulence factors of PA14 but was able to promote *C. elegans* survival (Li et al., 2014). Elastase or lasB is a metalloproteinase secreted by *P. aeruginosa*, with multiple roles leading toward cytotoxicity and degradation of host immune system (Kipnis et al., 2006). *C. elegans* exposed to LasB-knockout PA14 survived longer as compared to the normal virulent PA14 strain in the slow killing assay (Zhu et al., 2015). Intriguingly, our results demonstrate that actinomycete extract resulted in an increase of elastase production. Elsewhere, fatty acids have been shown to stimulate levels of elastase in *P. aeruginosa* (Kwan et al., 2011). Despite the increased levels of elastase produced by PA14 in presence of the actinomycete extract, it is worth noting that this did not translate to higher worm kills.

*C. elegans* possess 10 lysozyme-like proteins (*lys-1* to *lys-10*), with several of them associated with host defense (Mallo et al., 2002). Among these, *lys-7* have been shown to be immune-specific, with RNAi mediated *lys-7* knockdown worms showing increased sensitivity to pathogen killing (Mallo et al., 2002; Nandakumar and Tan, 2008; Simonsen et al., 2011). It has been reported that PA14 suppresses *C. elegans* immunity by repressing the expression of *lys-7* (Evans et al., 2008). Our results showed that the hexane partition of *Streptomyces* sp. CCB-PSK207 boosted the level of *lys-7* which was weakened during PA14 infection. Restoration of repressed *lys-7* during infection have also been reported with other single compounds and plant extracts (Dharmalingam et al., 2012; Kong et al., 2014a; Li et al., 2014). Concomitantly, uninfected worms treated with *Streptomyces* sp. CCB-PSK207 partition also showed an increase expression of *lys-7*, giving direct evidence of the presence of bioactive compounds capable of inducing *lys-7*. Taking into consideration that PA14 compromise host immunity through the suppression of various host defense molecules including *lys-7*, our results indicate that the bioactive partition have the capacity to restore this deficiency, leading to a rescue from mortality (Evans et al., 2008). While numerous reports of compounds attenuating PA14 virulence have been isolated from marine actinomycetes, to our knowledge, this is the first report of marine actinomycete metabolites inducing immunity in an infected host. A possible outcome of the induced elastase levels in PA14 in presence of the actinomycete extract is the reciprocal increase in innate immune response of host, as seen in insects (Andrejko and Mizerska-Dudka, 2011). Therefore, besides inducing *lys-7* as the defense molecule during infection, we do not rule out the possibility of an alternate stimulus of innate immunity caused by higher elastase production by PA14. A slow killing assay involving infection of *C. elegans* with LasB-knockout PA14 will be useful to endorse this possible route (Zhu et al., 2015).

GC/MS analysis of the *Streptomyces* sp. CCB-PSK207 bioactive fraction showed the presence of methyl esters of several saturated fatty acids including tridecanoic acid, tetradecanoic acid, and hexadecanoic acid. Simultaneously, methyl esters of branched-chain tetradecanoic acid, 12-methyl and pentadecanoic acid 14-methyl were also present. Some of these fatty acids have previously been reported from *Streptomyces* sp. (González et al., 2005; Ser et al., 2016). Methyl ester of tridecanoic acid, tetradecanoic acid, hexadecanoic acid, pentadecanoic acid 14-methyl, and tetradecanoic acid 12-methyl have also been reported in *C. elegans* (Henry et al., 2016). Although the actual mechanism by roles these compounds protect *C. elegans* from PA14-induced mortality is still unclear, some hints as to their possible functions could be derived from literature pertaining to *C. elegans* immunity and fatty acids. Two 18 carbon unsaturated fatty acids, gamma-linolenic acid (GLA, C18:3n6) and stearidonic acid (SDA, C18:4n3) are pivotal for *C. elegans* defense against PA14 infection as these fatty acids regulate basal expression of immune-specific genes including *lys-*7 (Nandakumar and Tan, 2008). Interestingly, hexadecanoic acid methyl ester can be elongated endogenously by worms to stearic acid (C18:0), followed by further desaturation to generate GLA or SDA, which could speculatively restore *lys-7* in PA14 infected worms (Watts and Browse, 2002). Elsewhere,1,2-benzenedicarboxylic acid mono (2-ethylhexyl) ester (MEHP) is also reported to induce the activation of the mitogen-activated protein kinase p38 (p38 MAPK) pathway, a pivotal signaling mechanism in *C. elegans* innate immunity (Kim et al., 2002; Rakkestad et al., 2010). Obliteration of *nhr-49*, a known master regulator of lipid metabolism in *C. elegans* also resulted in higher susceptibility to pathogenic infection, giving indication to the importance of fatty acids and *C. elegans* innate immunity (Sim and Hibberd, 2016).

In conclusion, this present study revealed the rescue of PA14-infect *C. elegans* by metabolites from a locally isolated *Streptomycetes* species. We also showed that this process circumvented bactericial or anti-virulences mechanisms and instead, induced worm immunity. Lastly, we showed that the bioactive molecules responsible for these observations are fatty acid methy-esthers which could hypothetically stimulate expression of *lys 7*.

## Ethics statement

Standard Operating Procedures involving *C. elegans* and living modified organisms (LMOs) were approved by the Universiti Sains Malaysia Animal Ethic Committee (AECUSM) and the Institutional Biosafety Committee (UKKP).

## Author contributions

Overall approach of study was designed by AS-C. All authors were involved in designing of experiments and data analysis. SF performed all the experiments. All authors were involved in preparation of manuscript.

### Conflict of interest statement

The authors declare that the research was conducted in the absence of any commercial or financial relationships that could be construed as a potential conflict of interest.
