# Supplementary material for: A Marine Actinomycete Rescues Caenorhabditis elegans from Pseudomonas aeruginosa Infection through Restitution of Lysozyme 7
Source: Front Microbiol. 2017 Nov 16;8:2267. doi: 10.3389/fmicb.2017.02267 (PMC5696594; doi:10.3389/fmicb.2017.02267)
Supplement: Supplementary file 1 [file DataSheet1.docx]

**List of supporting information legends**

**Table S1.** Morphological characteristics of 18 actinomycetes isolated from marine sediment samples from Songsong Island, Malaysia on Starch Casein Agar (SCA).

**Table S2. Time to death for 50% of the worm population (TD50) in hours treated with crude extract.** A5 methanolic extract gives the highest TD50. * denotes statistically significance, p<0.05, *t-test* in comparison to untreated control. Data were representative of three independent experiments.

**Table S3. Time to death for 50% of the worm population (TD50) in hours treated with partitions.** A5 hexane partition gives the highest TD50 value. * denotes statistically significance, p<0.05, *t-test* in comparison to untreated control. Data were representative of three independent experiments.

**Table S4. Time to death for 50% of the worm population (TD50) in hours treated with A5 fractions.** Fraction A5HB gives the highest TD50 value. * denotes statistically significance, p<0.05, *t-test* in comparison to untreated control. Data were representative of three independent experiments.

**Figure S1. Morphology of A5 *Streptomyces* sp. CCB-PSK207.** Isolate grown on ISP2 agar plates incubated at 28±2°C for 14 days.

**Figure S2. GC-MS spectrum of fraction A5HB from *Streptomyces* sp. CCB-PSK207.** Spectrum was obtained from an Agilent 6890 fitted with a capillary column of 30 m x 0.25 mm x 0.25 µm (Agilent HP-5ms, USA).

**Supplementary tables and figures**

**Table S1.** Morphological characteristics of actinomycetes isolated from marine sediment on starch casein agar (SCA)

| Isolate | Colony characteristic | | | | | | | |
| --- | --- | --- | --- | --- | --- | --- | --- | --- |
|  | **Aerial mycelia** | **Substrate mycelia** | **Shape** | **Margin** | **Elevation** | **Appearance** | **Optical property** | **Size**  **(mm)** |
|  |  |  |  |  |  |  |  |  |
| A3 | White | Light orange | Round | Smooth | Pulvinate | Dry | Opaque | 2 |
| A5 | White | White | Round | Smooth | Flat | Dry | Opaque | 5 |
| A20 | White | Pink | Round | Curled | Flat | Dry | Opaque | 5 |
| A22 | White | Brown | Round | Curled | Flat | Dry | Opaque | 5 |
| A24 | White | White | Round | Curled | Flat | Glistening | Opaque | 5 |
| A26 | Brown | White | Round | Smooth | Flat | Glistening | Opaque | 6 |
| A30 | White | Colourless | Round | Curled | Flat | Dry | Opaque | 3 |
| A31 | White | Yellow | Round | Curled | Flat | Dry | Opaque | 6 |
| A38 | Grey | Yellow | Round | Smooth | Pulvinate | Powdery | Opaque | 3 |
| A39 | Brown | Brown | Round | Curled | Flat | Dry | Opaque | 5 |
| A40 | Grey | Yellow | Round | Concentric | Raised | Powdery | Opaque | 3 |
| A41 | None | Yellow | Round | Smooth | Raised | Mucoid | Opaque | 2 |
| A42 | Grey | Yellow | Round | Concentric | Raised | Powdery | Opaque | 6 |
| A43 | White | Yellow | Round | Smooth | Umbonate | Dry | Opaque | 3 |
| A45 | Olive | Yellow | Round | Concentric | Raised | Powdery | Opaque | 5 |
| A47 | None | Peach | Round | Smooth | Flat | Mucoid | Opaque | 2 |
| A48 | White | Yellow | Round | Smooth | Flat | Dry | Opaque | 3 |
| A50 | None | White | Round | Smooth | Flat | Dry | Opaque | 1 |

**Table S2. Time to death for 50% of the worm population (TD50) in hours treated with crude extract.** A5 actinomycetes crude methanolic extract gives the highest TD50 value. * denotes statistically significant, *p*<0.05, *t-test* in comparison to untreated control. Data were representative of three independent experiments.

| Crude extract | TD50 ± SD |
| --- | --- |
| Untreated control | 87.23 ± 1.85 |
| A3 | 105.00 ± 3.02* |
| A5 | 107.67 ± 3.03* |
| A20 | 83.61 ± 2.85 |
| A22 | 100.00 ± 2.01* |
| A24 | 80.03 ± 3.00 |
| A26 | 96.94 ± 1.95 |
| A30 | 67.57 ± 1.38 |
| A31 | 68.97 ± 1.40 |
| A38 | 94.99 ± 2.00 |
| A39 | 69.38 ± 1.29 |
| A40 | 70.19 ± 1.28 |
| A41 | 72.78 ± 1.45 |
| A42 | 85.20 ± 3.08 |
| A43 | 76.19 ± 1.77 |
| A45 | 75.06 ± 1.79 |
| A47 | 77.01 ± 2.42 |
| A48 | 76.80 ± 2.08 |
| A50 | 76.41 ± 2.56 |

**Table S3. Time to death for 50% of the worm population (TD50) in hour treated with A5 partition.** A5 hexane extract gives the highest TD50 value. * denotes statistically significance, *p*<0.05, *t-test* in comparison to untreated control. Data were representative of three independent experiments.

| Solvent extract | TD50 ± SD |
| --- | --- |
| Untreated control | 81.33±3.77 |
| Aquos | 71.10±2.43* |
| Butanol | 51.034±1.024* |
| Ethyl acetate | 81.55±2.68 |
| Hexane | 102.16±5.54* |

**Table S4. Time to death for 50% of the worm population (TD50) in hours treated with A5 fractions.** Fraction A5HB gives the highest TD50 value. * denotes statistically significance, p<0.05, *t-test* in comparison to untreated control. Data were representative of three independent experiments.

| Fraction | TD50 ± SD |
| --- | --- |
| Untreated control | 61.33±3.82 |
| A5HA | 72.00±4.25* |
| A5HB | 93.60±3.85* |
| A5HC | 52.80±4.29 |

**
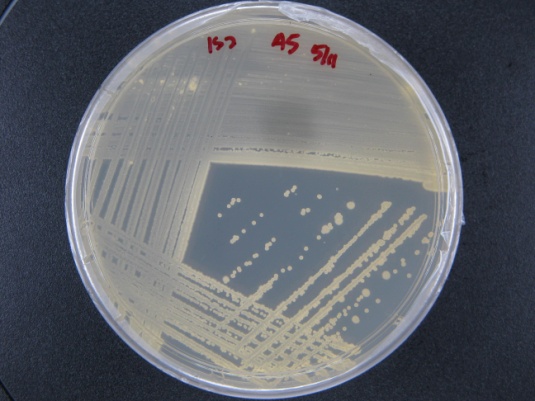

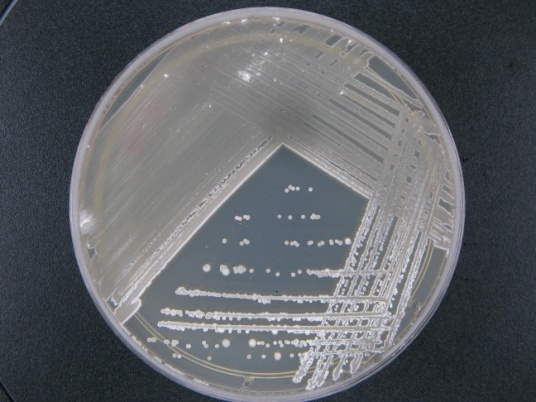
**

**Figure S1. Morphology of *Streptomyces* sp**. **CCB-PSK207** grown on ISP2 agar plates incubated at 28±2°C for 14 days.

**
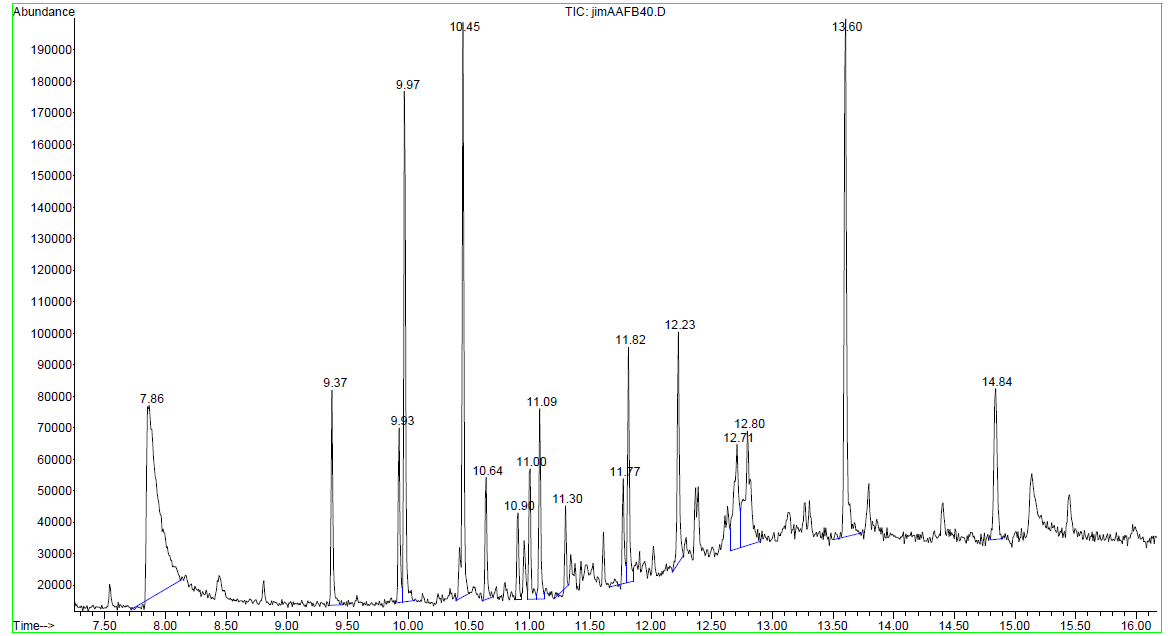
**

**Figure S2. GC-MS spectrum of fraction A5HB from *Streptomyces* sp. CCB-PSK207.** Spectrum was obtained from an Agilent 6890 fitted with a capillary column of 30 m x 0.25 mm x 0.25 µm (Agilent HP-5ms, USA).
